# The Effect of β-Carotene Supplementation on the Pharmacokinetics of Nelfinavir and Its Active Metabolite M8 in HIV-1-infected Patients

**DOI:** 10.3390/molecules17010688

**Published:** 2012-01-12

**Authors:** Nancy L. Sheehan, Rolf P. G. van Heeswijk, Brian C. Foster, Humayoun Akhtar, Neera Singhal, Isabelle Seguin, Lina DelBalso, Marc Bourbeau, Bobby M. Chauhan, Mohammed-Rachid Boulassel, David M. Burger, Richard G. Lalonde, Donald William Cameron

**Affiliations:** 1 Faculty of Pharmacy, Universite de Montreal, C.P. 6128, succursale Centre-Ville, Montreal, H3C 3J7, Canada; 2 Immunodeficiency Service, McGill University Health Centre, 3650 St. Urbain, Montreal, H2X 2P4, Canada; Email: lina.delbalso@muhc.mcgill.ca (L.D.); rachid.boulass@muhc.mcgill.ca (M.-R.B.); richard.lalonde@muhc.mcgill.ca (R.G.L.); 3 Division of Infectious Diseases, University of Ottawa at the The Ottawa Hospital/Research Institute, 501 Smyth Road, Ottawa, K1H 8L6, Canada; Email: rvheesw1@its.jnj.com (R.P.G.H.); neerasinghal@hotmail.com (N.S.); iseguin@ottawahospital.on.ca (I.S.); 4 Office of Science Laboratory, Therapeutics Products Program, Health Canada, 0900C2, Ottawa, K1A 0K9, Canada; Email: brian.foster@hc-sc.gc.ca (B.C.F.); chauhanbm@yahoo.com (B.M.C.); 5 Faculty of Medicine, Cellular and Molecular Medicine Department, University of Ottawa, 451 Smyth Road, Room 3206, Ottawa, K1H 8M5, Canada; 6 Agriculture and Agri-Food Canada, 93 Stone Road West, Guelph, N1G 5C9, Canada; Email: humayoun.akhtar@agr.gc.ca; 7 The University of Ottawa at the Ottawa Hospital/Research Institute, 501 Smyth Road, Ottawa, K1H 8L6, Canada; Email: marc_bourbeau@hotmail.com; 8 Department of Pharmacy & Nijmegen Institute for Infection, Inflammation and Immunity (N4i), Radboud University Nijmegen Medical Center, Geert Grooteplein 10, 6525 GA Nijmegen, The Netherlands; Email: d.burger@akf.umcn.nl

**Keywords:** β-carotene, HIV, nelfinavir, interaction, pharmacokinetics

## Abstract

β-Carotene supplements are often taken by individuals living with HIV-1. Contradictory results from *in vitro* studies suggest that β-carotene may inhibit or induce cytochrome P450 enzymes and transporters. The study objective was to investigate the effect of β-carotene on the steady-state pharmacokinetics of nelfinavir and its active metabolite M8 in HIV-1 infected individuals. Twelve hour nelfinavir pharmacokinetic analysis was conducted at baseline and after 28 days of β-carotene supplementation (25,000 IU twice daily). Nelfinavir and M8 concentrations were measured with validated assays. Non-compartmental methods were used to calculate the pharmacokinetic parameters. Geometric mean ratios comparing day 28 to day 1 area under the plasma concentration-time curve (AUC_0–12 h_), maximum (C_max_) and minimum (C_min_) concentrations of nelfinavir and M8 are presented with 90% confidence intervals. Eleven subjects completed the study and were included in the analysis. There were no significant differences in nelfinavir AUC_0–12 h_ and C_min_ (−10%, +4%) after β-carotene supplementation. The M8 C_min_ was increased by 31% while the M8 AUC_0–12 h_ and C_max_ were unchanged. During the 28 day period, mean CD4^+^ % and CD4^+^:CD8^+^ ratio increased significantly (*p* < 0.01). β-carotene supplementation increased serum carotene levels but did not cause any clinically significant difference in the nelfinavir and M8 exposure.

## 1. Introduction

Micronutrient deficiencies occur commonly in persons with human immunodeficiency virus (HIV) infection and are associated with poorer prognosis, although the role of micronutrient supplementation in the clinical management of HIV/AIDS remains controversial [1]. Still, complementary and alternative therapy use is common in the HIV-1-infected population. Over 65% of HIV-1-infected patients surveyed reported the use of herbs, vitamins or dietary supplements [2]. The supplementation of β-carotene, a carotenoid with provitamin A (retinol) activity, is of particular interest as below normal plasma carotene levels were found to be present in 31% of the HIV-infected population [3]. Low carotenoid levels in HIV infection may be caused by general malabsorption, fat malabsorption or altered metabolism and are of concern as carotene depletion may be associated with oxidative stress [4,5]. However, the effect of carotenoid supplementation in the HIV-infected population yielded conflicting results [6,7]. One study showed a reduction in mortality in patients on antiretroviral (ARV) treatment [6] whereas another study in patients without ARV treatment showed no added value of carotenoid supplementation in regards to AIDS-related mortality [7].

Though potentially beneficial, natural health products (NHP) such as β-carotene may interact with ARVs. An *in vitro* study suggested that *trans*-β-carotene had an inhibitory effect on cytochrome P450 (CYP) isoenzymes 2C9, 2C19 and 3A4 [8]. If this is confirmed *in vivo*, β-carotene could enhance the pharmacokinetics of protease inhibitors (PIs), non-nucleoside reverse transcriptase inhibitors (NNRTIs) and maraviroc, primarily by CYP3A4 inhibition, thereby increasing their efficacy as well as their concentration-related adverse drug reactions. Furthermore, increased ARV levels due to a carotene interaction could explain the apparent beneficial immunologic effects of carotene supplementation in patients on ARV therapy [6]. In contrast, an *in vitro* study has shown that β-carotene and retinol activates the pregnane-X-receptor (PXR) reporter gene in HepG2 cells, increasing the expression of CYP3A4/7, CYP3A5, multidrug resistance protein 1 and multidrug resistance associated protein 2 similarly to rifampicin [9], a drug known to induce the metabolism of PIs such as nelfinavir [10]. Possible induction of PI, NNRTI and maraviroc metabolism by β-carotene may result in suboptimal concentrations and foster the development of viral resistance. Thus, disparate *in vitro* findings warrant *in vivo* investigation, as a substance may simultaneously be a substrate, inhibitor and inducer of CYP, and net effects at steady-state *in vivo* may not be a reflection of isolated effects *in vitro*.

Nelfinavir is a PI primarily metabolised by CYP2C19 to its equally active metabolite M8 (hydroxy-*t*-butylamidonelfinavir), which is in turn metabolised by CYP3A4 to inactive metabolites [11,12]. We chose nelfinavir to conduct this *in vivo* drug-NHP interaction study to evaluate the effects of β-carotene supplementation on both CYP2C19 and CYP3A4. The primary objective of our study was to investigate the effects of β-carotene supplementation for 28 days (50,000 IU daily) on the steady-state plasma pharmacokinetics of nelfinavir and its active metabolite M8 in HIV-1-infected individuals.

## 2. Results and Discussion

Fifteen subjects signed the written informed consent and underwent the screening process from 2003 to 2005. One subject was excluded as he was concomitantly receiving nevirapine, a potent CYP3A4 inducer, and another subject was excluded after the protocol amendment as he was a smoker. Another subject chose to withdraw from the study before the first pharmacokinetic sampling day. Thus, twelve subjects were included and completed the study. Upon analysis of the medication administration diaries, all subjects reported to be adherent to nelfinavir and β-carotene during the 28 day study period, except for one subject who admitted having missed several doses of each. This subject’s results were removed from the analysis and the data presented are for eleven subjects. 

The demographics, virological, and immunological characteristics at baseline and at day 28 are shown in Table 1. The mean age was 45 years and the majority of subjects were male Caucasians. Weight and body mass index did not significantly change during the 28 day study period. The concomitant nucleoside reverse transcriptase inhibitors (NRTIs) consisted of zidovudine and lamivudine (n = 5; 45.4%), stavudine and lamivudine (n = 3; 27.3%), abacavir and lamivudine (n = 1; 9.1%), zidovudine, abacavir and lamivudine (n = 1; 9.1%) and stavudine, abacavir and lamivudine (n = 1; 9.1%). The mean duration on the nelfinavir-based ARV regimen was 3.9 years. 

Supplementation with β-carotene 25,000 IU twice daily resulted in a significant increase in serum carotene levels. The mean β-carotene content per capsule at the beginning (2003) and end of the study (2005) was respectively 28% (mean 31,966 IU; range 24,470–40,471) and 17% (mean 29,181 IU; range 27,074–32,015) more than the 25,000 IU claimed content by the manufacturer. As compared to the result in 2003, a 9% degradation over a two-year period occurred thereby confirming the stability of the β-carotene formulation that was used in the current study.

**Table 1:** Study population characteristics at baseline and after 28 days of β-carotene supplementation, 25,000 IU twice daily (n = 11).

	Baseline characteristics	Day 28 characteristics (n = 11)	*p* ^†^ value
	(n = 11)		
Age (years)	45.5 ± 9.4	-	-
Gender [n, (%) male]	9 (81.8)	-	-
Weight (kg)	76.6 ± 15.7	76.0 ± 16.6	0.489
Body mass index (kg/m^2^)	26.2 ± 4.0	26.1 ± 4.1	0.83
Ethnicity [n, (%)]			
- Caucasian	6 (54.5)	-	-
- African / African-American	4 (36.4)	-	-
- Asian	1 (9.1)	-	-
CD4^+^ (cells/μL)	616.2 ± 229.3	667.4 ± 330.5	0.258
CD4^+^ %	29.6 ± 8.5	33.3 ± 10.3	0.0009
CD8^+^ (cells/μL)	859.5 ± 435.1	759. 5 ± 413.0	0.091
CD8^+^ %	39.9 ± 13.2	37.8 ± 12.8	0.07
CD4^+^:CD8^+^ ratio	0.9 ± 0.6	1.1 ± 0.6	0.003
Absolute lymphocyte count (×10^9^/L)	2.0 ± 0.7	2.0 ± 0.7	0.873
n (%) with HIV viral load <50 copies/mL	10 (90.9)	10 (90.9)	-
	(1 patient had 63)	(1 patient had 50)	
Carotene level (μmol/L)	3.35 ± 1.29	5.16 ± 1.61	0.0056
(reference range: 1.0–5.5 μmol/L)			

Unless specified otherwise, data presented as mean ± standard deviation; ^† ^Paired t-test.

The median (range) pharmacokinetic parameters of nelfinavir and M8 on day 1 and day 28 are shown in Table 2.

**Figure 1:**
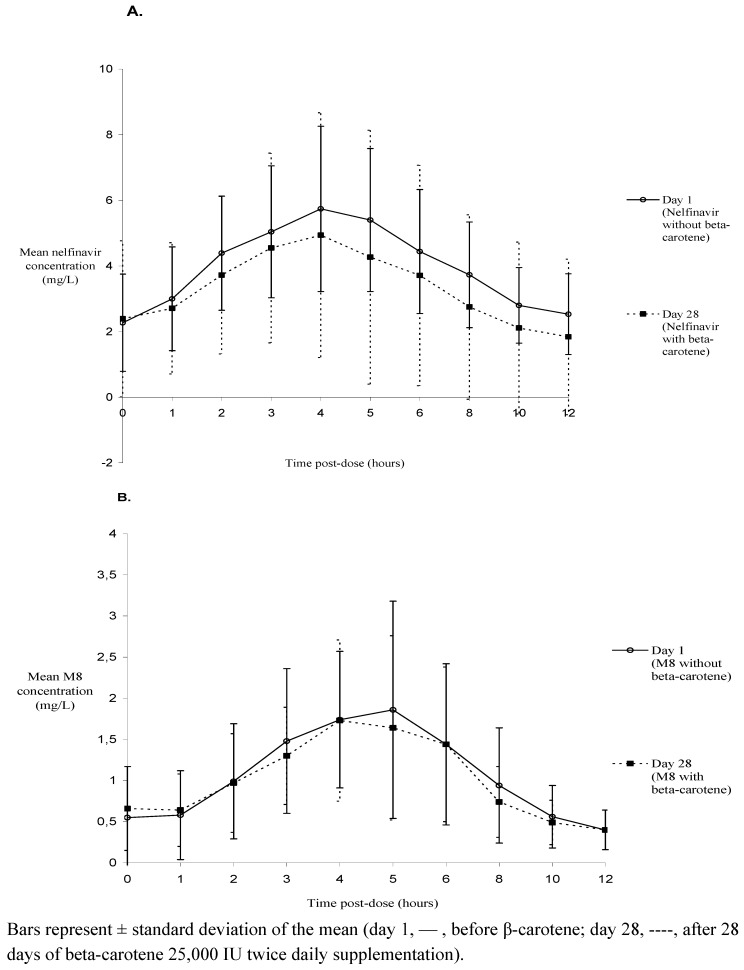
Mean nelfinavir (A, n = 11) and M8 metabolite (B, n = 9) plasma concentrations (mg/L) before and after 28 days of β-carotene supplementation.

**Figure 2:**
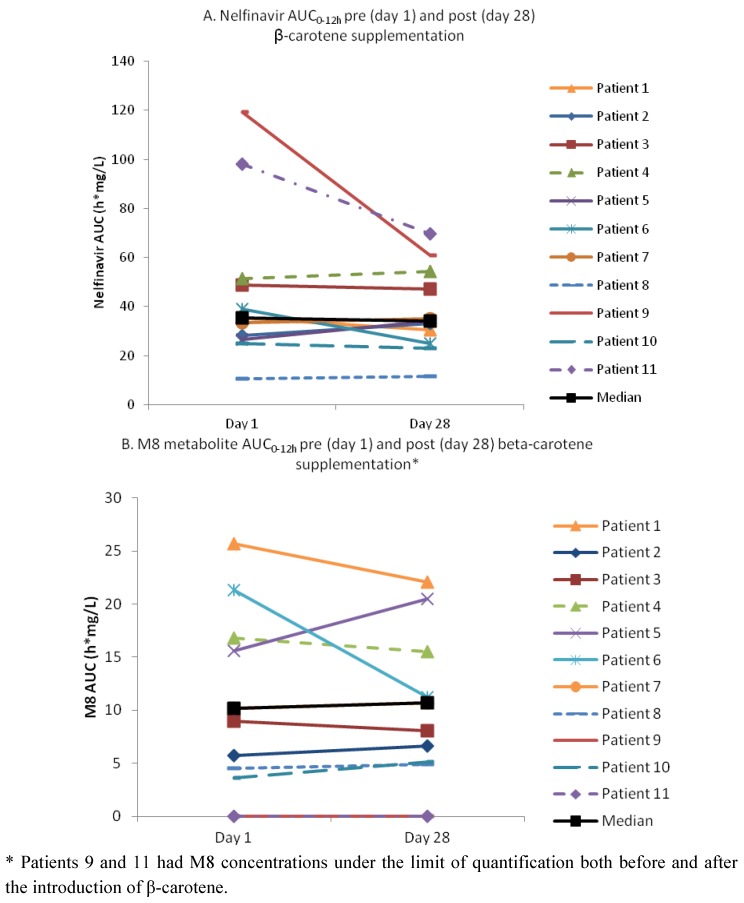
Individual steady-state area under the plasma concentration-time curve from 0 to 12 hours post-dose (AUC_0–12 h_) of nelfinavir and its M8 metabolite at baseline and after 28 days of β-carotene supplementation (25,000 IU twice daily) *.

Figure 1 shows the mean (± standard deviation) plasma nelfinavir (1a) and M8 (1b) concentrations over a twelve-hour period before and after 28 days of β-carotene supplementation. Based on the geometric mean ratio (GMR) and the 90% confidence intervals (CI), no clinically significant differences in nelfinavir and M8 exposure (area under the plasma concentration-time curve from 0 to 12 hours post-dose or AUC_0-12 h_) were apparent. However, there was a small decrease in nelfinavir AUC_0–12 h_ (−10%) and maximum concentration (C_max_) (−4%) and a small increase in nelfinavir minimum concentration (C_min_) (+4%) after β-carotene supplementation. The large 90% CI demonstrates large inter-subject variation. Furthermore, the effects were not consistent within the study population with some patients showing increases while others decreases in nelfinavir and/or M8 exposure (Figure 2).

Two subjects were excluded from the M8 and metabolic AUC ratio analysis as their M8 plasma concentrations were undetectable both before and after β-carotene administration. The GMR for the M8 C_min_ showed a 31% increase, whereas the M8 AUC_0–12 h_ was not significantly altered. No clinically significant differences were noted for the metabolic AUC ratio. 

The serum carotene concentration on day 28 did not correlate with day 28 nelfinavir or M8 AUC_0–12 h_, C_max_ or C_min_ parameters or with day 28/day 1 ratios of these parameters (all *P* values were greater than 0.3).

All but one of the eleven subjects included in the analysis had an undetectable HIV RNA viral load before and after β-carotene supplementation. This subject had a baseline and day 28 viral load of 63 and 50 copies/mL, respectively. The mean individual (± standard deviation) increase in CD4^+^ T lymphocytes was 75.1 ± 129.5 cells/μL. This difference was not statistically significant. The increases in the mean CD4^+^ % and CD4^+^:CD8^+^ ratio, however, were significantly greater after 28 days of β-carotene supplementation (*p* = 0.0009 and 0.003, respectively). The absolute lymphocyte count remained unchanged. No other significant laboratory changes, including renal and hepatic function, were documented. 

In general, β-carotene was well tolerated. Only one subject developed mild adverse reactions (occasional headaches and altered taste) possibly related to β-carotene as these symptoms appeared the day β-carotene was introduced and resolved after the end of β-carotene supplementation. No grade 2, 3 or 4 adverse events were noted. Though carotenemia or carotoderma is a known effect of carotenoid supplementation in persons without HIV infection [13], none of the subjects complained of yellow-orange skin discoloration.

**Table 2:** Steady-state plasma pharmacokinetic parameters of nelfinavir and its M8 metabolite at baseline and after 28 days of β-carotene supplementation (25,000 IU twice daily).

Pharmacokinetic parameter ^∆^	Nelfinavir alone (day 1)	Nelfinavir and Beta-carotene (day 28)	GMR and 90% CI
**Nelfinavir (n = 11)**			
AUC_0–12 h _(h*mg/L)	35.46 (10.40–119.36)	34.11 (11.37–69.61)	0.90 (0.81–1.05)
C_min_ (mg/L)	1.35 (0.10–7.73)	1.43 (0.20–2.41)	1.04 (0.68–2.38)
C_max_ (mg/L)	5.20 (2.08–13.57)	4.87 (2.03–10.34)	0.96 (0.87–1.11)
T_max_ (h)	4.00 (2.00–5.00)	4.00 (3.00–12.00) ^¥^	
t_½_ (h)	4.81 (2.65–14.40)	4.24 (2.51–8.83)	
**M8 (n = 9 ** **^§^** **)**			
AUC_0–12 h_ (h*mg/L)	10.18 (3.59–25.72)	10.71 (4.91–22.05)	0.99 (0.88–1.18)
C_min_ (mg/L)	0.30 (0.05–0.62)	0.34 (0.09–0.54)	1.31 (0.90–2.05)
C_max_ (mg/L)	1.53 (0.49–4.64)	1.87 (0.95–3.56)	1.02 (0.86–1.28)
T_max_ (h)	4.00 (3.00–5.00)	4.00 (3.00–12.00) ^¥^	
t_½_ (h)	2.72 (1.90–5.77)	3.21 (1.96–4.55)	
**Metabolic AUC ratio (n = 9 ** **^§^** **)**			
AUC M8: AUC			
nelfinavir	0.33 (0.14–0.73)	0.31 (0.17–0.72)	1.00 (0.90–1.14)

Nelfinavir 1250 mg twice daily given with a standardized breakfast (627 kcal, 42% fat); ^∆^ Data are reported as median and range; ^§^ Two subjects were excluded from the M8 and metabolic ratio analysis as M8 was always under the limit of quantification; ^¥^ Based on the plasma concentrations we suspect that subject 10 took his evening nelfinavir dose before the end of the 12 hour intensive pharmacokinetic day on day 28; Abbreviations: GMR, geometric mean ratio day 28 to day 1; CI, confidence interval; AUC_0-12 h_, area under the plasma concentration-time curve from 0 to 12 hours post-dose; C_min_, minimum plasma concentration; C_max_, maximum plasma concentration; T_max_, time post-dose of maximum plasma concentration; t_½_, plasma elimination half-life.

### Discussion

Overall, β-carotene supplementation did not have a significant influence on the AUC_0–12 h_ of nelfinavir, hence implying no clinically significant *in vivo* inhibition or induction of CYP2C19 or intestinal transporters. The M8 C_min_ GMR was increased by 31% but this effect was not consistent in all subjects and the M8 AUC_0–12 h_ was not significantly changed; thus, CYP2C19 induction or CYP3A4 inhibition (two mechanisms that could explain increases in M8) is unlikely. Our results show a large inter-patient variability with a possible inconsistent effect of β-carotene on nelfinavir and M8 AUC_0–12 h_ and C_min_. A few individuals had significant decreases in nelfinavir AUC after β-carotene supplementation but this was not accompanied by virologic failure within the 28 day study period. The pharmacokinetic parameters for nelfinavir and M8 in our study are comparable to historical cohorts [12,14].

The β-carotene 50,000 IU (30 mg) daily dose in our study was chosen based on the β-carotene dose received in a past randomized trial in the HIV-infected population [7]. The Canadian HIV Trials Network (CTN 091/CRIT Carotenoids Study Group) studied the influence of β-carotene supplementation on HIV morbidity and mortality and chose a daily β-carotene dose of 120,000 IU (72 mg). Due to β-carotene instability, however, by the end of the trial the β-carotene capsule content had reduced by 87% and some subjects had received only 15,000 IU (9.2 mg) daily [6]. To ensure that the same impediment was not occurring during our study, we measured β-carotene capsule content throughout the study and found that the β-carotene content was actually 17–28% greater than the claimed content by the manufacturer. Over a two-year period, the β-carotene content was relatively stable, with less than 10% decrease. The β-carotene capsule content at the end of the study still allowed a daily dose above our target daily dose of 50,000 IU. Although *in vitro* activation of PXR by β-carotene is known to be concentration-dependent in the range of 0.01–10 μM, little is known concerning the best plasma *in vivo* β-carotene concentration to study inhibition and induction of metabolic pathways and transporters [9]. 

In our study, β-carotene supplementation caused a 54% increase in serum carotene concentration after 28 days while that in the CTN 091 study doubled after six months [6]. Despite the same β-carotene supplement dose, the change in carotene concentration over the study period varied greatly amongst subjects from a 14% decrease to a 211% increase in carotene serum levels. Interpatient variability in β-carotene pharmacokinetics has been described. Some factors that can influence β-carotene bioavailability and metabolism are dietary fat, vitamin A status, dietary vitamin A and E intake, the food source or matrix of dietary β-carotene and genetic variability in proteins and transporters involved in β-carotene absorption and conversion to vitamin A [15,16,17,18,19,20]. Though subjects were instructed not to take any vitamin supplements two weeks prior to the start of the study and during the 28 day study period, other than their prescribed study β-carotene, we did not control for vitamin or fat intake originating from the diet. Our results show, however, that it is unlikely that the carotene concentration interpatient variability may have impacted on nelfinavir and M8 concentrations as no correlation was found between carotene day 28 serum levels and the pharmacokinetic results.

Two subjects in our study had undetectable M8 concentrations both before and after β-carotene supplementation. One of these subjects originated from Western Africa while the other was from the Philippines. Unfortunately, no pharmacogenetic analysis to evaluate CYP2C19 polymorphisms was available. The absence of M8 in these subjects favours the hypothesis that they may have been CYP2C19 poor metabolizers. The frequencies of CYP2C19 poor metabolizers in Zimbabwe and Ethiopia are 4 and 5.2%, respectively [21,22]. It is unclear whether CYP2C19 poor metabolizer frequency is similar in Western Africa. Twenty-three percent of the Filipino population has been described as being CYP2C19 poor metabolizers [23]. CYP2C19 681GA genetic polymorphisms, in particular the AA homozygote allele, increases nelfinavir exposure and virologic response [24]. Acquired CYP2C19 deficiency secondary to chronic liver disease may also decrease nelfinavir clearance and consequently decrease M8 formation [25]. None of the subjects in our study, however, had liver disease.

Though two laboratories analyzed the nelfinavir and M8 plasma concentrations, we do not believe this to be a limitation to our study. First, both these assays have been successfully validated by an external inter-laboratory quality control program for antiretroviral therapeutic drug monitoring [26]. In addition, this could not influence our GMR and 90% CI results as the concentrations on days 1 and 28 for a given patient were measured in the same laboratory and each subject’s plasma concentrations and pharmacokinetic parameters were paired for the statistical analysis; that is, each subject was compared to him/herself pre and post β-carotene supplementation. We recognize that the sample size is a limitation of the study, particularly given the large inter- and intra-patient variation in plasma drug concentrations. The study population was less than the planned sample size of twelve due to one subject’s non-adherence to the study and HIV treatment medications. At the end, the study may have lacked a sufficient sample size to show a significant difference in nelfinavir or M8 exposure.

β-Carotene supplementation with 50,000 IU per day for 28 days also resulted in a significant increase in the CD4^+^ % and CD4^+^:CD8^+^ ratio in our subjects none of which had a baseline carotene deficiency. Though not statistically significant, we also noted an increase in the absolute CD4^+^ lymphocyte count. We acknowledge that our immunologic findings must be interpreted with caution as this study had no control group and was not designed specifically for this objective. The results of numerous studies, some including a control group, are consistent however with our findings [6,27,28,29,30,31,32] and suggest that the increase in CD4^+^ lymphocytes produced by beta-carotene supplementation is dose or exposure dependent. The probability that T lymphocyte changes might have been due to background continuous immune reconstitution on effective anti-HIV treatment is low in our opinion, as the effect was clinically significant and the study interval was brief. Furthermore, the study subjects were already on stable, effective therapy with suppressed plasma viremia in all but one patient. This clinical benefit must be weighed against potential risks in smokers, former smokers and workers exposed to asbestos. In these populations, beta-carotene supplementation has been shown to increase the relative risk of lung cancer, cardiovascular disease-associated mortality, lung cancer-associated mortality and overall mortality [33].

## 3. Experimental

### 3.1. Study Design

This was a multicentre open-label non-randomized steady-state pharmacokinetic study in HIV-infected individuals already receiving nelfinavir 1,250 mg twice daily as part of their antiretroviral regimen. Participating sites were the Ottawa Hospital (Ottawa, Canada) and the Montreal Chest Institute (McGill University Health Centre, Montreal, Canada). Eligible subjects on stable effective therapy including nelfinavir 1,250 mg twice daily were asked to co-administer oral β-carotene 25,000 IU (equivalent to 15 mg) twice daily for four weeks. Plasma concentrations of nelfinavir and M8 were measured for twelve hours after intake of nelfinavir before (day 1) and after four weeks (day 28) of β-carotene supplementation. 

### 3.2. Subjects

HIV-infected adults were eligible to participate if they were receiving nelfinavir 1,250 mg twice daily with at least two NRTIs for at least two weeks. Other inclusion criteria included having signed a written informed consent, having no evidence of an acute illness, using adequate contraception in female patients of childbearing potential, and willing to discontinue all natural health products two weeks prior to day 1.

Patients were excluded if they were using other PIs or NNRTIs, were pregnant or breastfeeding, had abnormal haematology or biochemistry laboratory results at screening (haemoglobin < 85 g/L, absolute neutrophil count < 1000 cells/μL, platelet count < 50,000 /μL, aspartate aminotransferase, alanine aminotransferase or total bilirubin > 3 times the upper limit of normal, creatinine or lipase > 1.5 times the upper limit of normal), had acute or chronic renal, liver or pancreatic disease, had an active AIDS-defining illness or malignancy, were receiving a medication other than nelfinavir known to inhibit or induce CYP2C19 or CYP3A4-mediated metabolism, or had a condition that may impede medication or study protocol adherence.

Half-way through enrolment, concern for potential risk of β-carotene supplementation in smokers to increase lung cancer and associated mortality was raised, and a protocol amendment excluding smokers was approved by the research ethics boards [33]. Subjects who smoked and who had already completed the study before the amendment were not excluded from the data analysis.

### 3.3. Study Drugs

All subjects were instructed to take nelfinavir 1,250 mg (five 250 mg tablets) and 1 capsule of β-carotene every 12 hours with food. β-Carotene supplementation was given to subjects from the same lot (Exact^TM^, Pharmetics Inc., Laval, Canada, DIN 01904388). As per the manufacturer, each capsule contained 25,000 IU β-carotene USP. As β-carotene content in capsules is known to be sensitive to degradation, the content of the capsules was quantified at the beginning and end of the study. The β-carotene content was measured by a previously published validated high performance liquid chromatography (HPLC) assay [34] and is reported as the percentage of the manufacturer’s claimed content.

### 3.4. Assessment and Data Abstraction

The screening visit included a review of the subjects’ medical chart, complete medication history, physical examination, baseline haematology, biochemistry, virology (HIV RNA) and immunology tests (CD4^+ ^and CD8^+ ^T lymphocytes) as well as hepatitis B and C screening. Women also underwent a urine pregnancy test. On the pharmacokinetic sampling days pre and post β-carotene supplementation (days 1 and 28, respectively), serum carotene levels, HIV RNA viral loads (lower limit of detection 50 copies/mL) and CD4^+^ and CD8^+^ T lymphocytes (flow cytometry) were measured. A physical examination was also repeated at day 28. The vital signs, list of concomitant medications and adverse drug reactions were recorded at each study visit. A diary was given to each patient to record all dates and times of nelfinavir and β-carotene intake between day 1 and 28. 

### 3.5. Pharmacokinetic Sample Collection

On days 1 and 28, subjects arrived at the clinic early in the morning, fasting and before their morning nelfinavir dose. Pharmacokinetic sample collection was started in the morning for all patients in order to avoid the effects of diurnal variation of nelfinavir concentrations [35]. An intravenous catheter was placed for serial blood draws that were done just before the morning nelfinavir dose and at 1, 2, 3, 4, 5, 6, 8, 10 and 12 hours post ingestion of nelfinavir 1,250 mg. A standardized breakfast (627 calories, 42% fat) was given with the morning nelfinavir dose. Subjects were instructed not to take their evening nelfinavir dose before the end of the 12 hours sampling period. Blood samples were collected in sodium heparinised 6 mL tubes. Plasma was isolated by centrifugation (1,550 *g* × 10 minutes) and stored in cryotubes at less than −20 °C until batch analysis of nelfinavir and M8 plasma concentrations was conducted.

### 3.6. Analytical Methods of Nelfinavir and M8

The nelfinavir and M8 plasma concentrations were measured in two laboratories due to logistical difficulties. Plasma nelfinavir and M8 concentrations for the first seven subjects, pre and post β-carotene supplementation, were analyzed by a liquid chromatography with tandem mass spectrometry (LC-MS/MS) method at The Ottawa Hospital (Ontario, Canada) [14]. The within-day and between-day coefficients of variation for both nelfinavir and M8 were reported to be less than 12% [14]. The remaining samples were analyzed by HPLC at the Radboud University Nijmegen Medical Centre (Nijmegen, the Netherlands). The within-day and between-day coefficients of variation in this laboratory varied between 3.4–4.0% and 0–4.9% for nelfinavir and 1.7–5.3% and 1.0–4.3% for M8 [36]. Both these assays have been successfully validated by an external inter-laboratory quality control program [26]. 

### 3.7. Pharmacokinetic Analysis

Non-compartmental methods using WinNonLin Pro Version 4.0 (Pharsight Corporation, Cary, USA) were used to analyze plasma concentration versus time data of nelfinavir and M8. The highest and lowest observed plasma concentrations were defined as C_max_ and C_min_, respectively, with the corresponding sampling time post-dose at the time of maximum concentration as T_max_. The elimination rate constant (*k*_el_) was determined by an analysis using least squares linear regression of at least 3 data points in the elimination phase. The plasma elimination half-life (t_1/2_) in hours was calculated by the equation t_1/2_ = ln2/*k*_el_. The AUC_0-12 h_ was estimated using the linear trapezoidal rule. The metabolic AUC ratio was calculated by dividing the AUC_0–12 h_ of M8 by the AUC_0–12 h_ of nelfinavir for each patient. 

### 3.8. Statistical Analysis

Baseline and day 28 demographic and clinical data are reported as means (±standard deviation) or as proportions. The paired-t test was used to compare day 1 to day 28 subject characteristics. A *P* value less than 0.05 is considered statistically significant. Pharmacokinetic parameters are reported as medians with ranges; medians are used to describe the pharmacokinetic parameters as with small sample sizes they are less sensitive to extreme values which may be caused by interpatient pharmacokinetic variability. As is recommended for the comparison of pharmacokinetic parameters in drug interaction studies, geometric means of the ratios (GMR) comparing day 28 to day 1 AUC_0–12 h_, C_max_ and C_min_ of nelfinavir and M8 for each individual patient were calculated and are presented with 90% confidence intervals (CI) [37,38]. A GMR with 90% CI was also calculated for the metabolic AUC ratio. As per the Food and Drug Administration guidelines for the analysis and interpretation of drug-drug interaction studies, a default 80 to 125% no effect boundary was used to determine the clinical significance of the potential interaction [38]. When the 90% CI of the GMR is below 0.8 or above 1.25 the interaction is considered clinically significant. The effect of β-carotene on the pharmacokinetics of nelfinavir and M8 was considered not clinically significant (equivalent to a lack of interaction) if the 90% CI of the GMR for the AUC was entirely within the no effect boundary range of 80–125%. Non parametric correlation analyses between day 28 carotene serum levels and the nelfinavir and M8 AUC_0–12 h_, C_max_ and C_min_ as well as the day 28/day 1 ratios of these pharmacokinetic parameters were conducted using the Spearman’s rank test. Statistical analyses were performed using SPSS for Windows, version 13.0 (SPSS Inc, Chicago, USA). 

### 3.9. Ethical Considerations

The study protocol and amendment were approved by the research ethics boards at The Ottawa Hospital and the Montreal Chest Institute. All subjects signed a written informed consent before the screening process was started and were free to withdraw from the study at any time.

## 4. Conclusions

We conclude that β-carotene supplementation with 25,000 IU twice daily did not cause a clinically significant net change in nelfinavir and M8 steady-state exposure in the overall study population. To our knowledge, this is the first prospective *in vivo* drug—NHP interaction study focusing on the effects of β-carotene on the metabolism of a medication. The results from this study are helpful as they allow us to put forward the hypothesis that β-carotene at this dose may not interact significantly with other medications metabolized by CYP2C19 and/or CYP3A4. Prospective drug—NHP interaction studies with other CYP2C19 and CYP3A4 substrates are needed to confirm these results. Finally, our results are also relevant for future β-carotene supplementation studies in HIV as they suggest that any immunological benefits are unlikely to be secondary to significant increases in antiretroviral concentrations. 
